# Detection of the Most Common Microorganisms and Their Resistance against Anti-microbials in Intubated Patients in an ICU in Kerman, Iran

**Published:** 2010

**Authors:** Farhad Sarafzadeh, SeyedMojtaba Sohrevardi, Maryam Gharehghozli, Mehdi Ahmadinejad

**Affiliations:** a *Pharmaceutics Research Center and Faculty of Medicine, Kerman University of Medical Science, Kerman, Iran.*; b *Kerman Neuroscience Research Center and Faculty of Pharmacy, Kerman University of Medical Science, Kerman, Iran.*; c *Faculty of Medicine, Kerman University of Medical Science, Kerman, Iran*

**Keywords:** Minimum inhibitory concentration, Antimicrobial resistant, Intensive care unit, Intubated patients, *Klebsiella*

## Abstract

The purpose of this study was to determine the prevalence of anti-microbial resistance in intubated patients in intensive care unit (ICU) of Bahonar hospital in Kerman province, Iran during the year 2008.

Tracheal samples were obtained from 111 intubated patients in the ICU by broncoalveolar lavage method. Amikacin, Ceftazidim and Imipenem were used to evaluate antibiotic susceptibility. For detecting anti-microbial susceptibility, minimum inhibitory concentration method were used. Colony counts equal or more than 104 microorganisms/mL were considered resistant.

Overall we obtained positive tracheal cultures from 32 patients (29%) out of 111 intubated ones. The most common micro organisms isolated were *Klebsiella *(90.6%), *Acinetobacter *(28.1%) and *Pseudomonas *(21.9%). The results showed that the most common resistance was against to ceftazidim. The susceptibility of *Klebsiella*in tracheal cultures to the antibiotics was only 5%. *E. coli *in both sexes was 100% resistant to the tested antibiotics.

In the ICU, There was a very big problem concerning antibiotic resistance. Most of the isolated microorganisms were resistant to both the old and the new antibiotics. It may be related to the inappropriate use of antibiotics, bacterial contamination of enteral feeding and infection transmission by medical staff or instruments.

## Introduction

Anti-microbial resistance is an increasingly important consideration when initializing empiric anti-microbial therapy in intensive care unit (ICU). Infection with a resistant organism has been associated with increased morbidity and mortality as well as increased hospital cost. The unique nature of ICU environment makes this part of the hospital a focus for the emergence and spread of many anti-microbial resistance pathogens. Rate of resistance has been increased for most pathogens associated with hospital acquired infections among ICU patients and rates are almost universally higher among ICU patients than non-ICU patients (1, 2).

Studies have indicated that nosocomial infection occurred in 5-10% of all hospitalizations in Europe, North America and in more than 40% of hospitalizations in parts of Asia, Latin America and sub-Saharan Africa (3).

Drug abuse, miss diagnosis, length of stay in ICU, not being disposable of suction tubes, reusing suction tubes for one patient and bacterial contamination of enteral feeds are the causes that increase anti-microbial resistance. Enteral feedings provide a favorable medium for exponential growth of microorganisms (4).

Contaminated feedings has increased the risk of nosocomial infections such as diarrhea, pneumonia and septicemia (5). The sources of enteral tube feeding contamination are:

1- ingredients such as non-sterile water, raw fruits and vegetables.

2- personnel factors such as inadequate hand washing, touch contamination and preparation of feeding by personnel with respiratory infection. 

3- formula manipulation and handling such as dilution and reconstitution.

4- environmental factors including improper ventilation and air dusts (6).

The hospital wards with the highest prevalence observed were the ICU, both neonatal and adult (accounting for 88.8% of infection) (7).

**Table 1:** Percentage of anti-microbial resistance by type of antibiotics

**Microorganism**	**Imipenem**			**Amikacin**			**Ceftazidim**		
	R%	SR%	S%	R%	SR%	S%	R%	SR%	S%
*Klebsiella*	65	30	5	65	30	5	95	0	5
*Acinetobacter*	66.6	33.4	0	66.6	33.4	0	66.6	0	33.4
*Pseudomonas*	0	100	0	33.4	66.6	0	66.6	0	33.4
*E. coli*	0	100	0	100	0	0	100	0	0
*Alcaligenes*	50	50	0	50	50	0	95	0	5
*S. aureus*	100	0	0	100	0	0	100	0	0

R: Resistant; SR: Semi-resistant; S: Susceptible

It is not more than two decades that *Acinetobacter *has emerged as an important nosocomial pathogen and hospital outbreaks caused by this organism have increased worldwide (8). The purpose of this study was to determine the prevalence of anti-microbial resistance in intubated patients in the ICU of Bahonar hospital in Kerman province, Iran, during the year 2008. 

## Experimental

*Patients*

485 patients were admitted to the ICU from March 2008 until March 2009. 45 out of them were excluded from the study because of lack of data and lab results. 

*Sample collection*

Tracheal samples were obtained from 111 intubated patients out of 440. Samples were collected by broncoalveolar lavage (BAL) method and were cultured in blood agar and EMB in 37 °C for 24 h. 

Anti-microbial susceptibility was performed by minimum inhibitory concentration (MIC) method. For this purpose, the broth micro dilution method was utilized. MIC end points were defined as the lowest concentration of antibiotic that resulted in no bacterial growth as indicated by the absence of turbidity (9-10). 

Amikacin, ceftazidim and imipenem was used to evaluate antibiotic susceptibility. MIC ≥ 64 demonestrated resistance and MIC ≤ 16 showed susceptibility to Amikacin. MIC ≥ 32 and MIC ≤ 8 was demonstrated for resistance and susceptibility to ceftazidim, respectively. These amounts for imipinem were MIC ≥ 16 and MIC ≤ 4.

*Statistical analysis*

Statistical analysis was performed using SPSS (ver. 13). The obtained data were analyzed with an independent sample t-test. A p-value less than 0.0001 was considered statistically significant. Colony count ≥ 104 microorganism/mL was considered positive for tracheal cultures (11).

## Results

Out of 111 intubated patients, there were 25 women (22.5%) and 86 men (77.5%). The average age for men and women was 33.7 ± 19.6 and 41.5 ± 20.6, respectively.

Overall we obtained positive tracheal culture for 32 patients (29%) and negative tracheal culture for 79 (71%) out of 111 intubated ones. among positive ones 25 patients were male and 7 were female. The most common microorganisms isolated were *Klebsiella *(90.6%), *Acinetobacter *(28.1%), *Pseudomonas *(21.9%), *E. coli *(12.5%), *Alcaligenes *(6.3%), *S. aureus *(3.1%) and *Enterobacter *(0%), respectively.

Isolated *Klebsiella *was resistant to ceftazidim (95%), and its resistancy to amikacin and Imipenem were 65% (Table 2). The results showed that the most common resistancy was related to ceftazidim. The susceptibility of *Klebsiella *to the antibiotics in tracheal cultures was only 5%. 

The most effective antibiotic for *Pseudomonas *in tracheal cultures was ceftazidim. *E. coli *was 100% resistant to ceftazidim and amikacin. *Entrobacter *wasn’t seen in both genders. Among women and men the most common microorganism was *Klebsiella*. *E. coli *in both sexes was shown most resistant to antibiotics (100%). *Acinetobacter*in females was shown 100% resistant to imipenem and ceftazidim (Table 2- 4). 

**Table 2:** Percentage of microorganism in positive tracheal cultures by type of gender (n = 32, 25 male)

**Sex **	***Klebsiella***	***Acinetobacter***	***Pseudomonas ***	***E. coli ***	***Alcaligenes***	***S. aureus***
Female (%)	19	2	4	4	3	2
Male (%)	43	30	20	7	11	5

**Table 3:** Percentage of anti-microbial resistance to antibiotics in women

**Antibiotic **		***Klebsiella***	***Acinetobacter***	***Pseudomonas ***	***E. coli ***	***Alcaligenes***	***S. aureus***
Imipenem	R SR S	81.2 18.8 0	100 0 0	75 25 0	33.3 66.7 0	100 0 0	100 0 0
Amikacin	R SR S	56.2 43.8 0	0 100 0	75 25 0	75 25 0	50 50 0	50 0 50
Ceftazidim	R SR S	100 0 0	100 0 0	100 0 0	100 0 0	100 0 0	100 0 0

R: Resistant; SR: Semi-resistant; S: Susceptible

**Table 4:** Percentage of anti-microbial resistance to antibiotics in men

**Antibiotic **		***Klebsiella***	***Acinetobacter***	***Pseudomonas ***	***E. coli ***	***Alcaligenes***	***S. aureus***
Imipenem	R SR S	71.4 24.4 4.2	71.4 28.6 0	85.7 14.3 0	71.4 28.6 0	54.5 36.3 9.2	83.3 0 16.7
Amikacin	R SR S	61.2 34.6 4.2	78.5 17.8 3.7	58.3 25 16.7	57.1 42.9 0	69.2 23 7.8	40 60 0
Ceftazidim	R SR S	93.8 4.2 2	92.8 0 7.1	90.4 4.8 4.8	100 0 0	72.7 9 18.3	100 0 0

R: Resistant; SR: Semi-resistant; S: Susceptible

The most frequent length of stay in ICU was 8 to 30 days (43.1%). During the first 48 h of admission there was no evidence of *Enterobacter *and *S. aureus *(Table 5). The percentage of anti-microbial resistance based on length of hospital stay is presented in Table 6. 

**Table 5:** Frequency of microorganisms based on length of hospital stay

**length of stay**	***Klebsiella***	***Acinetobacter***	***Pseudomonas***	***E. coli***	***Alcaligenes***	***S. aureus***
< 48 h	58.3	25	8.3	8.3	25	0
3-7 days	36.7	30	26.7	13.3	16.7	3.3
8-30 days	56.7	20	18.3	6.7	11.7	11.7
> ? 30 days	66.7	27.8	44.4	22.2	5.6	0

**Table 6:** Percentage of anti-microbial resistance to antibiotics by duration of hospitalization

			***Klebsiella***	***Acinetobacter***	***Pseudomonas***	***E. coli***	***Alcaligenes***	***S. aureus***
< 48 h	Imipenem	R SR S	85.7 14.3 0	66.6 33.4 0	100 0 0	100 0 0	0 100 0	- - -
	Amikacin	R SR S	28.5 71.5 0	100 0 0	0 100 0	100 0 0	50 50 0	- - -
	Ceftazidim	R SR S	100 0 0	100 0 0	100 0 0	100 0 0	100 0 0	- - -
3-7 days	Imipenem	R SR S	80 20 0	77.7 22.3 0	85.7 14.3 0	0 77.7 22.3	100 0 0	100 0 0
	Amikacin	R SR S	90 10 0	88.8 11.2 0	37.5 37.5 25	66.6 33.4 0	80 20 0	0 100 0
	Ceftazidim	R SR S	90 10 0	100 0 0	85.7 14.3 0	100 0 0	100 0 0	100 0 0
8-30 days	Imipenem	R SR S	71.7 23 5.3	75 25 0	90.9 9.1 0	50 50 0	42.8 42.8 14.4	83.3 0 16.7
	Amikacin	R SR S	56.2 37.5 6.3	58.3 33.3 8.4	81.8 9.1 9.1	25 75 0	57.1 14.4 14.4	50 33.3 16.7
	Ceftazidim	R SR S	93.8 3.1 3.1	91.6 0 8.4	90.9 0 9.1	100 0 0	57.1 28.7 28.7	100 0 0
> 30 days	Imipenem	R SR S	83.3 16.7 0	50 50 0	66.6 33.4 0	50 50 0	100 0 0	- - -
	Amikacin	R SR S	75 25 0	50 50 0	62.5 25 12.5	100 0 0	100 0 0	- - -
	Ceftazidim	R SR S	100 0 0	75 0 25	100 0 0	100 0 0	100 0 0	- - -

R: Resistant; SR: Semi-resistant; S: Susceptible

## Discussion

Kumari *et al. *in India studied on 370 patients in ICU. The highest mean resistance was to cefazolin (98.8%) and ampicillin (97.6%) while the lowest one was to amikacin (48.5%) (12).

Another study on anti-microbial resistance among gram-negative organisms in ICU in USA showed that the increased prevalence of extended spectrum β-lactamases has contributed to the finding of multi-drug resistance among bacterias such as *Klebsiella*and *E. coli *(13). 

It seems that anti-microbial resistance among nosocomial pathogens depends on the site of infection or the type of microbiologic specimen (14). In another research in Spain, no resistance to beta-lactams and levofloxacin was found (15). 

In Shanghai in 2003, the resistance of *S. pneumonia *was 81%. 51% of isolated had intermediate and high level penicillin resistances. 58% were resistant to ampicillin, 6.6% to cefazolin, 6.6% to ceftriaxone, 85.7% to erythromycin, 66.7% to chindamycin and 28.2% to chloramphenicol (16). 

In ICU, there is a very big problem concerning antibiotics resistance. Ampicillin and the first generation of cephalosporins have been never used to treat infection in the ICU. But from above studies we find that these medicines are still used in ICU. 

In 2007 in Turkey, imipenem-resistant *Acinetobacter baumannii*was isolated from 60 (53.7%) patients (17). In the present study it was found that 29% of intubated patients had positive tracheal cultures and *klebsiella*was found in 90.6% of culture positive patients. Also *Acinetobacter*was more resistant to imipenem in this study (66.6%) than in Turkey’s.

Patterson *et al. *evaluated the use of imipenem after substitution of ceftazidim with pipracillin/tazobactam, demonstrating no difference after changing of the antibiotics (18). 

Recorded data on bacteria isolated in 2000 in Brazil showed a high rate of Pseudomonas’ resistance to ceftazidime (37.5%) and imipenem (75%).

*Acinetobacter *spp. was also resistant to cefazidime in 93.2% of isolates (19). But, we showed that *Acinetobacter *was resistant to ceftazidim in 66.6% of isolates, *S. aureus *was 100% resistance to imipenem, amikacin and ceftazidim, and *E. coli *was 100% resistant to amikacin and ceftazidim. 

Overall, only isolated *Klebsiella *was shown 5% susceptibile to imipenem and amikacin and other microorganisms were not susceptible. 

We found that all isolated microorganisms were 100% resistant to ceftazidim in women. On the whole, microorganisms were shown to have more susceptibility to antibiotics in men than in women. Bacterial contamination of eternal feeding may have an important role in ICU infection (20). Here in Iran we usually don’t have access to standard enteral nutrition formula or they are too expensive for most patients and we have to use blenderized and homemade food for them. This kind of enteral nutrition is made by patient’s family member in a large amount and is stored in refrigerator in ICU for 24-28 h, thus is susceptibile to bacterial contamination. 

In first 48 h of patient’s stay in ICU, we had positive cultures for *Klebsiella*, *Acinetobacter*, *pseudomonas*, *E. coli *and *Alcaligenase*. It seems too dangerous. At the time of the study, we did not have single use airways for connecting patients to the ventilator machine, but we use it now and we think it helps us to decrease infection risks. Another interesting result was that all isolated microorganisms were resistant to ceftazidim during 48 h of admission. 

Effective strategies for prevention of anti-microbial resistance in ICU are: prevent, diagnose and treat infection effectively, use anti-microbial agents wisely, limit the unnecessary use of them and prevent transmission (21, 22). On the other hand, administration of standard enteral nutrition formula is better than homemade or blenderized feeding. Not disposable ventilator tubes are also an important factor in ICU. 

Panahi Y and Vessal G reported that *Klebsiella *and *Acinetobacter *were the most common isolated pathogen in the Sina and Shariati hospital in Tehran province and Iran, respectively (22, 23). Their results had similarity to our findings.

Drug and therapeutic committees (DTC) can help medical staff to choose antibiotics wisely in each field based on guidelines or protocols. We don’t have DTC in our hospital and it may worsen rational use of antibiotics. 
